# Impact of sustained health policy and population-level interventions on reducing the prevalence of obesity in the Caribbean region: A qualitative study from The Bahamas

**DOI:** 10.3389/fpubh.2022.926672

**Published:** 2022-08-30

**Authors:** Francis Poitier, Ricky Kalliecharan, Bassey Ebenso

**Affiliations:** Nuffield Centre for International Health and Development, University of Leeds, Leeds, United Kingdom

**Keywords:** health policy, obesity, The Bahamas, Caribbean, document review, policy analysis

## Abstract

**Background:**

The 2020 Global Nutrition Report highlights that despite improvements in select nutrition indicators, progress is too slow to meet the 2025 Global Nutrition Targets. While the Latin America and the Caribbean (LAC) region has achieved the greatest global reduction in undernutrition (stunting, underweight, and wasting) in the past decade, it also has the highest prevalence of people with overweight worldwide. Since the early 2000s, the region has mounted increasingly comprehensive and multi-sectoral policy interventions to address nutritional health outcomes. The Bahamas is one such LAC country that has used consistent policy responses to address evolving nutritional challenges in its population. After addressing the initial problems of undernutrition in the 1970s and 80s, however, overconsumption of unhealthy foods has led to a rising prevalence of obesity which The Bahamas has grappled with since the early 2000s.

**Objective:**

This study develops a timeline of obesity-related health policy responses and explores the macrosocial factors and conditions which facilitated or constrained public health policy responses to obesity in The Bahamas over a 20-year period.

**Methods:**

This multi-method case study was conducted between 2019 and 2021. A document review of health policies was combined with secondary analysis of a range of other documents and semi-structured interviews with key actors (policymakers, health workers, scholars, and members of the public). Data sources for secondary data analysis included policy documents, national survey data on obesity, national and regional newspaper websites, and the Digital Library of the Caribbean database. An adapted framework approach was used for the analysis of semi-structured interviews.

**Results:**

Between 2000 and 2019, a series of national policies and community-level interventions were enacted to address the prevalence of obesity. Building on previous interventions, obtaining multi-sectoral collaboration, and community buy-in for policy action contributed to reducing obesity prevalence from 49.2 to 43.7% between 2012 and 2019. There are, however, constraining factors, such as political and multi-sectoral challenges and gaps in legislative mandates and financing.

**Conclusion:**

Sustained multilevel interventions are effective in addressing the prevalence of obesity. To maintain progress, there is a need to implement gender-specific responses while ensuring accessibility, availability, and affordability of nutritious food for all.

## Introduction

The Global Nutrition Report of 2020 highlights that despite some improvements in select nutrition indicators, progress is too slow to meet the 2025 Global Nutrition Targets and that no country was on track to meet all 10 of them (1). At the same time, overweight and obesity are increasing rapidly in almost all countries, with little evidence of reduction. While the Latin America and the Caribbean (LAC) region has achieved the greatest global reduction in undernutrition (stunting, underweight, and wasting) in the past decade (2), ironically, the LAC region has the highest prevalence of people with overweight worldwide (3). Though the region has made progress in improving health indicators and in the accessibility, availability, and affordability of nutritious food (4), evidence shows that overweight and obesity have reached epidemic proportions in the region (2, 5). Some LAC countries also report a double burden of malnutrition in which stunting and/or micronutrient deficiencies coexist with overweight and obesity (2, 5).

In response to the above challenges, the Caribbean region has, since the early 2000s, mounted increasingly comprehensive and multi-sectoral policy and population-level interventions. The Declaration of Port-of-Spain on Uniting to Stop The Epidemics of Chronic Non-Communicable Diseases (NCDs) in 2007 recognised NCDs' threat to health and socioeconomic development (4). It builds on the Nassau Declaration of 2001, which recognised that “the health of the Region is the wealth of the Region”—a landmark acknowledgement that is said to underscore the importance of health to other development goals. Compared with other regions of the world, the Caribbean Heads of Governments were the first to hold a summit at that level which specifically focused on NCDs and is often credited as elevating the issue to the world stage (4, 6). The summit's declaration emphasised a multi-sectoral approach to preventing and controlling obesity and other NCDs by addressing unhealthy diets, physical inactivity, tobacco and alcohol abuse, and strengthening health services (7). The Declaration continues to inform regional policy and national responses (6).

The Bahamas is one such country in the Caribbean that has been using regional and global roadmaps, alongside consistent national policy responses, to address evolving nutritional challenges in its population. Like other Caribbean countries, The Bahamas has faced issues with undernutrition throughout its history. This is partly due to a beleaguered agricultural industry of scale and adverse climate events, such as hurricanes and droughts (8, 9). This has led to a heavy reliance on food imports from other countries, such as the United States (4). Other factors, such as globalisation, trade policies, and the growth of multinational food companies, have also impacted the Bahamian food landscape in various ways (10). For example, multinational fast food companies build brand loyalty through national media advertisements (11), community event sponsorships (12), and multiple convenient locations (13). These strategies lead to a proliferation of these companies and increased access to cheap processed foods, which contribute to malnutrition (14, 15). Despite these challenges, the country mounted successful policy and population interventions in the 1970s and 1980s to address undernutrition problems, including introducing price controls for food items, community health projects, health promotion interventions, and legislation.

**Figure 1:**
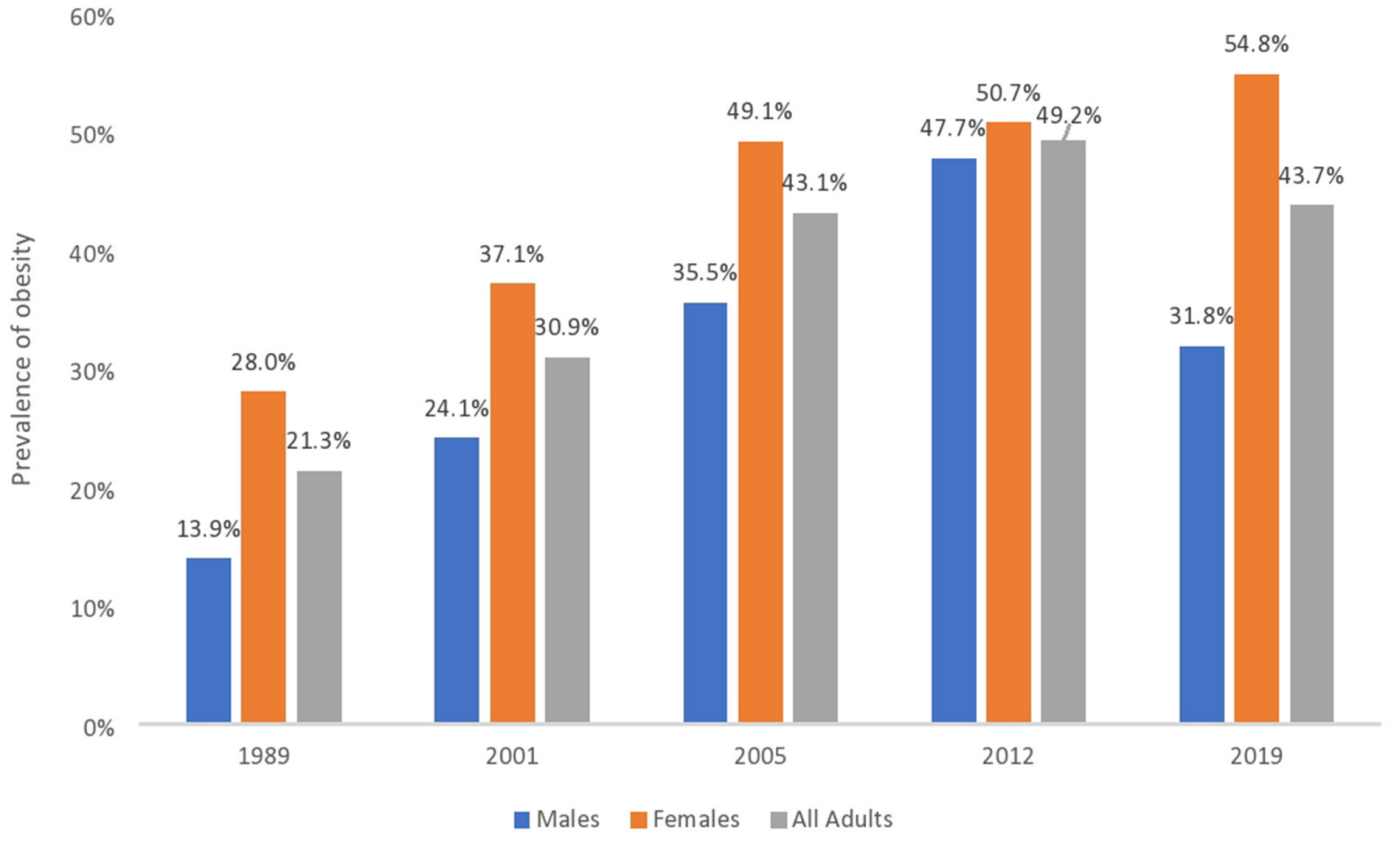
The prevalence of obesity in The Bahamas disaggregated by sex and year. Bahamian national reports show a consistent increase in the prevalence of obesity in adults between 1989 and 2012. It also shows a decrease in adult prevalence rates between 2012 and 2019. There is, however, a gendered health disparity. Prevalence rates continue to increase in women up to 2019 but have decreased in men between 2012 and 2019. The years listed represent the times when data were available from reports of Bahamian national surveys.

Since 1989 however, a new challenge has emerged due to the rising prevalence of obesity in the country (see Figure 1). Bahamian national reports reviewed show a consistent increase in obesity prevalence rates among all adults between 1989 and 2012; the rates were 21.3 and 49.2%, respectively. In 2019, however, the obesity prevalence rate among all adults was 43.7%, representing a 5.5 percentage point (pp) reduction (an 11.2% decrease) since 2012. This decline between 2012 and 2019 may mean a turning of trends in the prevalence of obesity in adults overall.

There is, however, a gender health disparity. Between 2005 and 2012, the prevalence of obesity among men rose by 12.2 pp (or a 34.4% increase), while it increased by 1.6 pp (or a 3.3% increase) in women during the same period. The prevalence among women continued to climb between 2012 and 2019, from 50.7 to 54.8%, representing a 4.1 pp increase (or a 7.5% increase). These data are in line with global figures where women have a higher prevalence of obesity (2, 16, 17). At the same time, the prevalence rate among men dropped by 15.9 pp (or a 33% decrease) from 47.7% in 2012 to 31.8% in 2019. This represents a reversal of previous trends seen up to 2012. According to national reports reviewed, men have been consistently more exposed to smoking and drinking (18–20), while women living with obesity are believed to be more compliant with professional advice (21). Other socio-cultural factors impact the prevalence of obesity along gender lines. These factors include cultural expectations about the body (22), beauty ideals (23), dietary practises (24), and finance (4). These indicate that social and public health policy alone may not affect obesity trends long term but can work as a composite with other interventions and socio-cultural and political-economic factors. While the rates of obesity have decreased in the country between 2012 and 2019, the policy responses that might be related to these changes need further exploration to better understand their individual and synergistic role in reducing the prevalence of obesity in The Bahamas.

Policy responses are actions and activities conducted or implemented to affect institutions and services of the public health system (25). They can include national policies (i.e., a set of mandated actions), population-level interventions (i.e., a set of actions targeting the whole population), or community engagement interventions (i.e., a set of actions targeting specific communities). Health policy responses can target core health system components (26). Some policy responses may not be written, which explains why Ward et al. [(27), p. 44] describe policies as being beyond a discrete entity or output, but a process that brings certain principles or ideas into practise. Policy responses must contend with wider factors in policy and health system environments which can facilitate or confine their implementation (28–31).

There are important insights to be learned about the public health policies, population- and community-level interventions mounted in The Bahamas, and the key enabling or constraining factors that shaped those policy responses. This paper, therefore, aims to: (1) develop a timeline of relevant obesity-related health policy responses between 2000 and 2019; and (2) explore the macrosocial factors and conditions which facilitated or constrained public health policy responses to obesity in The Bahamas.

## Materials and methods

### Study setting

The study occurred in The Bahamas. The Bahamas is a country made up of 700 islands and cays in the Atlantic Ocean. The country is approximately 50 miles (~80 km) from the United States.

**Figure 2:**
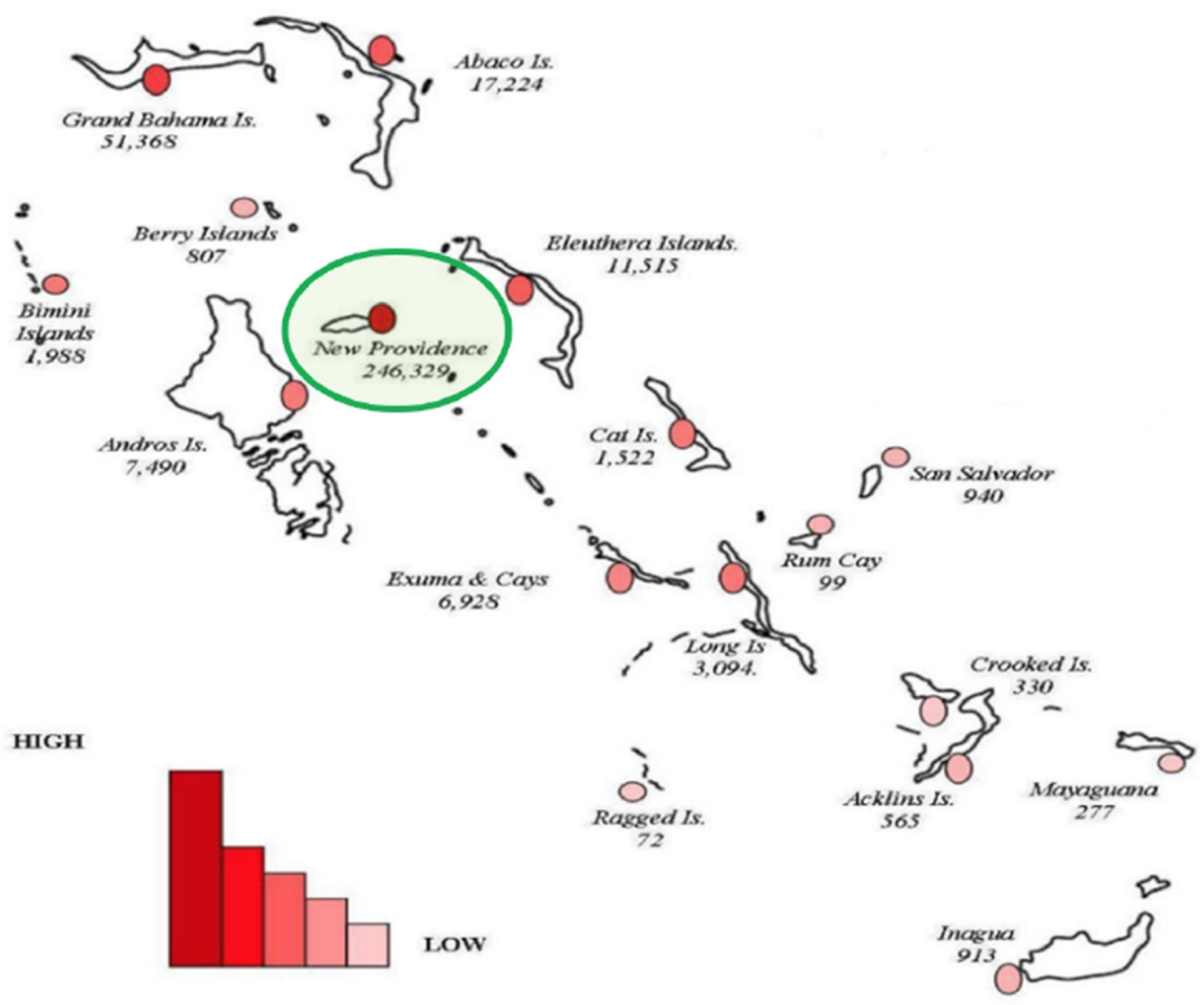
Map of The Bahamas showing the geographical and population spread with the sizes of population for inhabited islands in 2010. Adapted from BDOS (32). The capital island (New Providence) has the highest population of residents of the country (see green ring).

According to the 2010 Census (32), there are 351,461 residents of the country, the majority of which live in the nation's capital, New Providence (i.e., 246,329 residents) (see Figure 2). A new census is underway, but adverse climate events (such as Hurricane Dorian in 2019) and the coronavirus disease 2019 (COVID-19) pandemic caused significant delays.

### Study design

A multi-method, multi-phase case study was conducted between 2019 and 2021 in The Bahamas. Case studies, a form of qualitative research, are in-depth exploratory investigations to understand the contexts and processes of a research problem (33). This approach was selected to obtain rich, contextual, and detailed information to addressthe research aim.

The case study combined multiple data collection methods in three phases:

1. Document review of relevant government health policies and secondary analysis of survey data (of obesity prevalence)
2. Semi-structured interviews with key actors (policymakers, health workers, scholars, and members of the public)
3. Member checking, or respondent validation, interviews to enhance the trustworthiness of findings through the verification and assessment of the results in earlier research phases (34, 35). In this case, virtual interviews were conducted with participants to explore emerging findings and to provide an opportunity for additional data collection (34).

NVivo and the Microsoft Office suite were used for data management and storage.

### Document review

Document review, or document analysis, is a systematic procedure of reviewing or evaluating documents to develop empirical knowledge, elicit meaning, and gain understanding (36). Document review is a potential source of empirical data for case studies (36). It involves the finding, selecting, appraising, and synthesis of data contained in documents.

Documents were obtained from four main sources: (1) national health websites, such as the Ministry of Health of The Bahamas (MOH); (2) regional websites such as the Pan American Health Organization (PAHO); (3) the database of the Digital Library of the Caribbean; and (4) conversations with key stakeholders.

In reviewing these websites and databases, the search was narrowed specifically to The Bahamas and obesity between 1970 and 2019. This was due to The Bahamas gaining independence in 1973 and the aims of this research. The following inclusion criteria were applied to the search:

1. Local health documents, including policies, strategies, guidelines, and plans inclusive of draft versions
2. Documents related to an aspect of obesity prevalence (such as food, physical activity)
3. Accessible digitally or through correspondence with the MOH or recruited participants.

Documents were excluded if they could not be obtained within the project's data collection timeframe (i.e., up to December 2019).

Some regional health policy documents from the Caribbean were reviewed to provide contextual information on The Bahamas.

A list of the key documents reviewed is included in Supplementary material 1.

#### Data extraction and analysis of policy documents

Health policy documents were reviewed to identify and understand the government's response to obesity and how it may have evolved.

Examination of documents was done through an iterative process and constant comparison. Documents were initially scanned to determine their suitability and to understand each document's applicability to the research aim. If suitable, documents were then more thoroughly reviewed and summarised. Patterns between and within documents were highlighted through coding and recoding data with attention paid to similarities, differences, absences, sparseness, and incompleteness. Documents were reviewed critically, as documents may not be precise or complete with all the information or events that occurred [(36), p. 33]. Supplementary material was therefore ascertained through newspaper documents and semi-structured interviews. The document review helped to generate new questions for interviews and acted as a form of triangulation. Further data were extracted according to the framework below to provide a systematic process. Where available, extracted data included:

- Code and Name of the policy document
- Author/Agency publishing the document
- The date document was published
- Island locations mentioned
- Major themes/summary of activities and actions
- Contextual information
- Other policies mentioned
- Referred to In (i.e., whether the policy may have been referred to in other documents)
- Changes/Amendments to the policy
- Type of document (e.g., whether it was a survey, report, policy brief, programme evaluation, etc.)
- Funding source.

Quantitative information on the prevalence of obesity was noted for descriptive statistical analysis.

### Semi-structured interviews

Semi-structured interviews were conducted in two phases. Phase 1 interviews were conducted in person between October 2019 and December 2019 with 31 participants. This included 13 health workers, 11 members of the public, 4 policymakers, and 3 scholars. Phase 2 interviews (a set of 9 virtual member checking interviews), were conducted in August 2021 to enhance the trustworthiness of the findings of phase 1 interviews, fill initial gaps in research findings and provide opportunities for additional data (34, 35). Phase 2 interviews were virtual due to the ongoing COVID-19 pandemic.

Purposive and convenience sampling techniques were used for participant recruitment. Purposive sampling allows for the deliberate selection of participants appropriate for the study (35) while convenience sampling allowed for selecting participants that were most accessible under various conditions and circumstances (37).

Participants in this study were at least 18 years of age, able to communicate in English, and provide informed consent. Conversations took place in a private setting in public health facilities or places of the participant's choosing deemed suitable for interview.

Semi-structured interviews were audio-recorded, if permitted by participants, and transcribed verbatim. In cases where participants wished not to be recorded, detailed notes were taken. Notes were also taken during and after conversations to capture, *inter alia*, general themes, new ideas expressed, or strong opinions. Semi-structured interviews lasted an average of 34 min (range 10–70 min). Interviews with health workers were limited to a maximum of 15 min by health facility officials.

### Data analysis and triangulation

An adapted framework approach was employed to identify key themes from the semi-structured interviews. The framework approach is used to generate policy and practise-oriented findings while maintaining the integrity of individual participant responses (35). The framework approach involved familiarisation with the data, identification of key themes and concepts, indexing, charting, mapping, and interpretations (38, 39). The approach allowed for both themes identified through document review and those emerging from the interviews to be analysed. Data from document review and semi-structured interviews were triangulated to identify themes, meanings, and interpretations.

The document review and semi-structured interviews, therefore, worked together to address the research aims.

### Research ethics

This research was approved by research committees at the University of Leeds School of Medicine Research Ethics Committee (MREC-18-073) and in-country from the Public Hospital Authority/University of the West Indies Research Ethics Committee in The Bahamas (PHA/31/1-B-2).

## Results

The results are organised into two sections, each including themes and subthemes (see Table 1). The first section outlines the timeline of obesity-related health policy responses, and the second section notes key constraining and enabling factors influencing policy responses to obesity.

**Table 1:** Organisation of results according to a summary of findings.

**3.1. Timeline of relevant obesity-related health policy responses in The Bahamas**
*(1A) Relevant obesity related health policy responses in The Bahamas from 1970 to 2000*
*(1B) Timeline of relevant obesity-related health policy responses in The Bahamas since 2000*
*(1C) Content of health policy responses*
*(1D) Summary of key actions and activities*
1. Nutrition promotion
2. Community engagement
3. Combined approaches
4. Advocacy
5. Food quality
6. Other responses
**3.2. Key constraining and enabling factors influencing obesity responses**
*(2A) Notable constraints*
1. Political and multi-sectoral integration challenges
2. Gaps in legislative mandates
3. Need for monitoring implementation and updating policy
4. Funding and gaps in financial support
5. Sex and gender now coming into focus
*(2B) Notable enabling factors*
1. Building on consistent policy responses
2. Multi-sectoral support and collaboration
3. Buy-in for further action

### Timeline of relevant obesity-related health policy responses in The Bahamas

#### Relevant obesity-related health policy responses in The Bahamas from 1970 to 2000

We found an evolution of public health policy responses based on the priority nutritional health challenge at the time. Between 1970 and 1989, policy responses primarily focused on the issue of undernutrition in all age groups. In contrast, between 1990 and 2000, there was a focus on nutrition data gathering and national breastfeeding targeting babies aged less than 2 years. We found that after 2000, public health policy responses to obesity became increasingly sustained and comprehensive.

#### Timeline of relevant obesity-related health policy responses in The Bahamas since 2000

**Figure 3:**
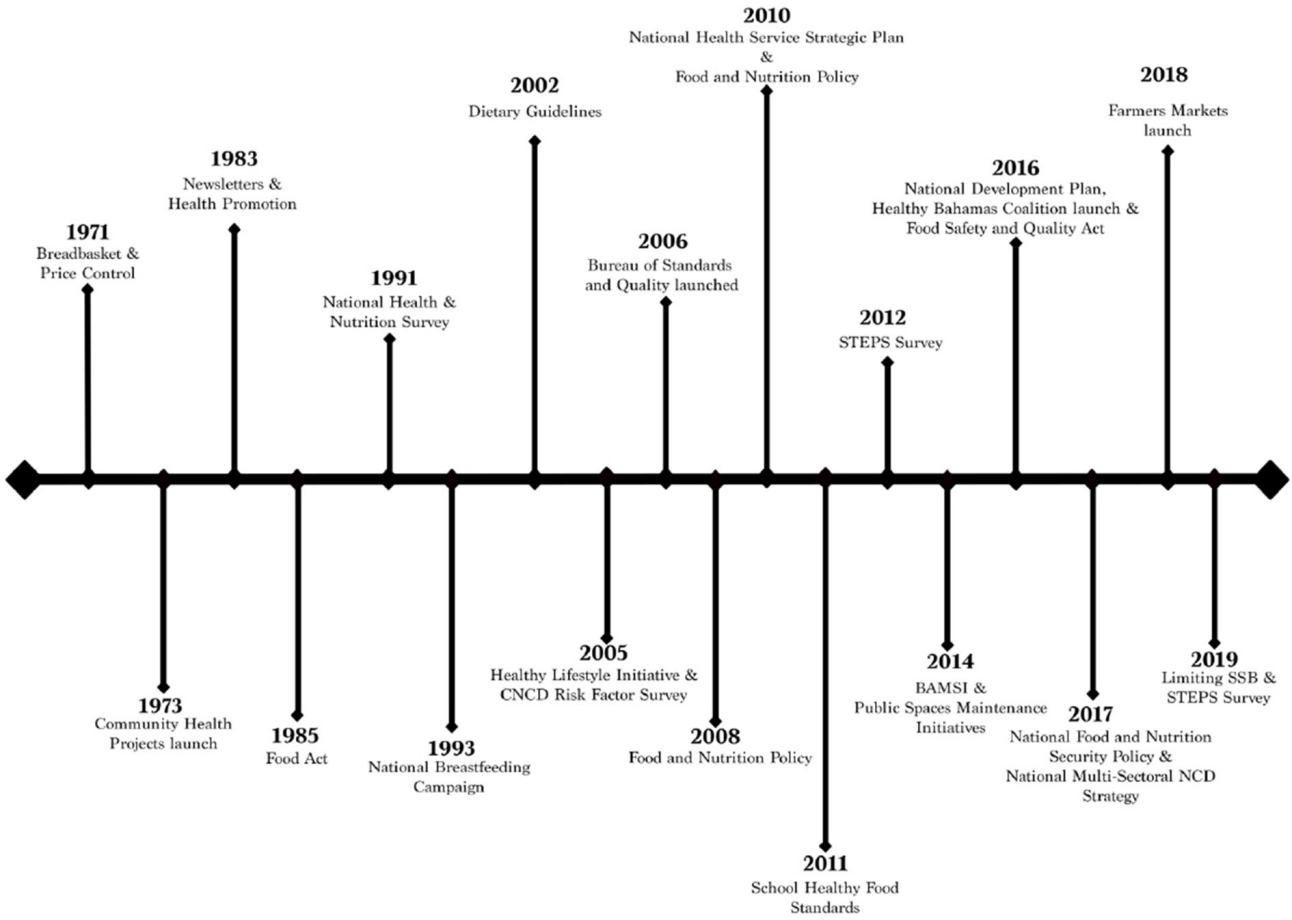
Timeline of policy responses to the prevalence of obesity in The Bahamas. The timeline shows a range of national policies and population- and community-level interventions implemented after the 2000s. In most instances, documents did not specify how long policies were in effect, with the exceptions of The National Health Services Strategic Plan (2010–2020), The National Food and Nutrition Security Policy (2017–2022), and the National Multi-Sectoral NCD Strategy (2017–2022).

We identified eight national policies and six population-level interventions implemented between 2000 and 2019 to address the prevalence of obesity in The Bahamas (see Figure 3).

The eight national policies initiated between 2000 and 2019 were: (i) the National Food-based Dietary Guidelines (2002), (ii) the National Food and Nutrition Policy (2008, 2010), (iii) the National Health Service Strategic Plan (2010), (iv) the School Healthy Food Standards (2011), (v) the Food Safety and Quality Act (2016), (vi) the draft National Development Plan (2016), (vii) The National Food and Nutrition Security Policy (2017), and (viii) the National Multi-sectoral NCD Strategy (2017).

We also identified six population-level interventions in the period, including (i) the launch of the Healthy Lifestyle Initiative (2005), (ii) the establishment of the Bureau of Standards and Quality of food and nutrition (2006), (iii) the Bahamas Agriculture and Marine Science Institute (2014), (iv) Public Spaces Maintenance Initiatives (2014), (v) the launch of the Healthy Bahamas Coalition (2016), and (vi) the launch of Farmers Markets (2018) to provide healthy food choices for the Bahamian population.

National policies and population-level interventions inspired targeted community engagement interventions to guide individual behaviour, including a ban on sugar-sweetened beverages in public schools and health facilities, workplace and school seminars, and mass public health education campaigns. Data gathering surveys (i.e., Risk Factor and STEPS Surveys) supported the policies and population-level interventions.

#### Content of health policy responses

This section offers a brief overview of the content of identified health policy responses.

Before 2000, health policy responses primarily focused on the undernutrition of the whole population as a priority public health issue. For example, Price Control legislation commenced on 30^th^ July 1971, establishing “control and regulation of the price of goods and services” in The Bahamas (40). The legislation sets the maximum price of essential items for community “wellbeing,” which became known as the Breadbasket Food Items list. These items aimed to address issues of undernutrition at the time and included products such as butter, cooking oil, mayonnaise, grits, cheese, flour, corned beef, and other items. According to a health worker:

“*See what happen is initially when the breadbasket was created in the 1970s…we had a different issue. The majority of the population was underweight. So at that time, your focus was to, pretty much, see how to feed a family of, let's say, 4–5. Make sure that they get sufficient nutrients and just weight gain. That was a different focus. Now we have the opposite problem. The majority of us overweight. Or obese. So those breadbasket items have to be revised because we don't have those same issues as we did some 30–40–50 years ago.”*

The health worker references the evolution of nutritional health challenges in the country and the need for updating policy responses to address the prevalence of obesity. According to national reports, there are “a wide range of programmes aimed at addressing undernutrition, and there are less in controlling overweight and obesity” [(41), p. 22]. In this way, The Bahamas is like other Caribbean countries, which similarly tackled undernutrition with some success, but now face a new reality of growing obesity prevalence (15).

**Figure 4:**
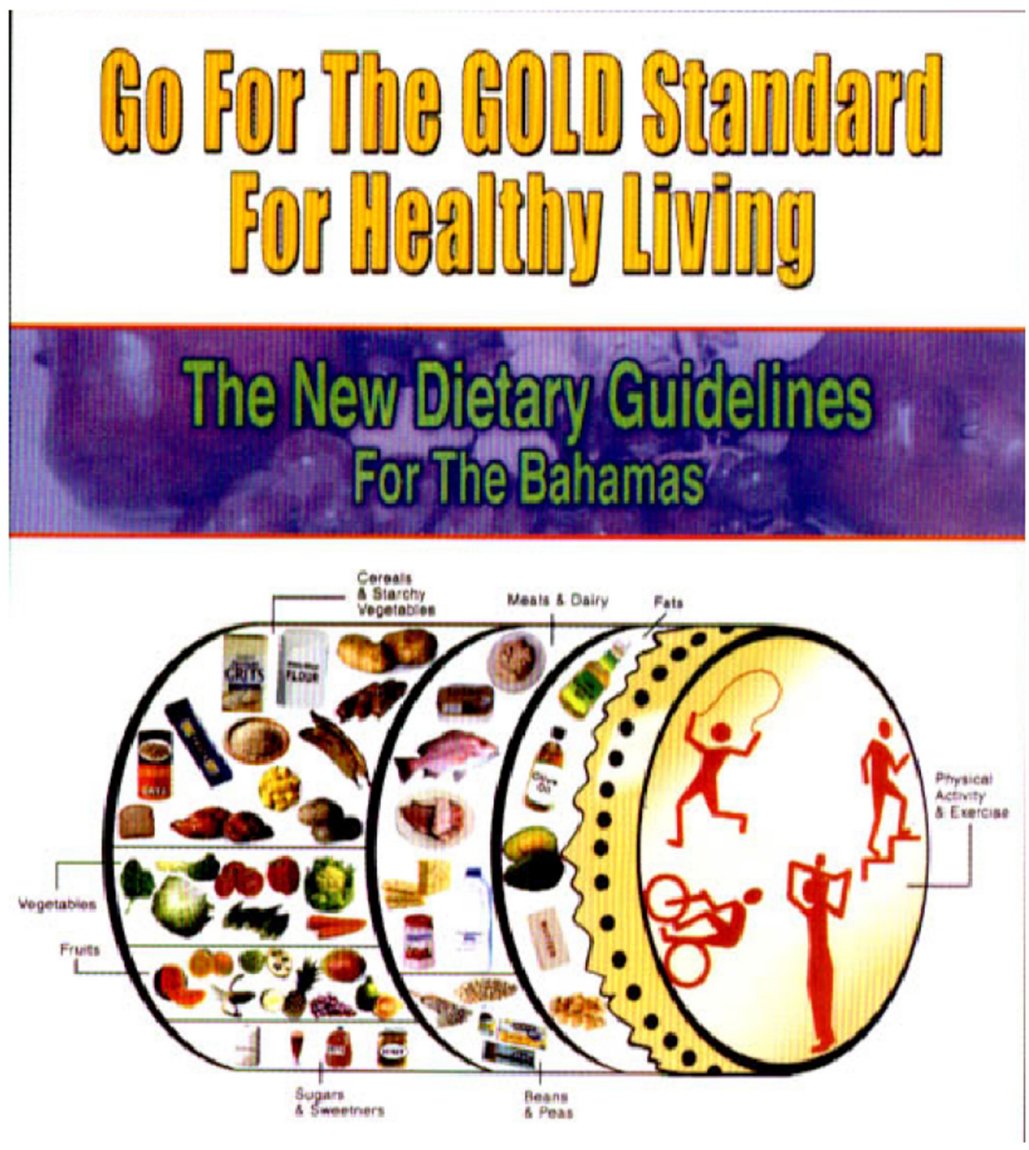
Bahamian food-based dietary guidelines developed in 2002. The image shows a culturally adapted version of the food pyramid using a goatskin drum used in the Bahamian festival, Junkanoo, adapted from BMOH (42).

In 2002, The Bahamas was the first country in the Caribbean region to develop a local Food-based Dietary Guideline. The Guidelines take the shape of a Bahamian goatskin drum—an item of cultural importance given its use in the national festival—Junkanoo (see Figure 4). It is an adaptation of the traditional dietary triangle used elsewhere. The Guidelines advocate for consuming various fruits and vegetables and choosing foods for nutritional value “not for the ‘name brand' or cost.” Such advocacy is indicative of the cultural context where fruits and vegetable intake is considerably low (18, 43) and may indicate a propensity for choosing foods based on brand name and cost over nutritional value.

In 2005, the country's focus on tying nutritional responses to healthy lifestyle interventions came to the fore with The Healthy Lifestyle initiative. It aimed to increase awareness of NCDs and reduce their impact (44). A policymaker mentioned that teams of nurses were organised through a Healthy Lifestyles Secretariat to survey different communities in The Bahamas. Every month, activities were organised to reach Bahamians in the workplace, school, or community (45). By 2016, the Initiative appears to have expanded as a Healthy Bahamas Coalition was formed with similar objectives, such as conducting strategic planning for health promotion programmes, advocacy, and addressing risk factors to NCDs, such as nutrition and physical activity.

In 2006, a Bureau of Standards and Quality was initiated. The Bureau aims to improve the quality of local food and standardise label requirements for fresh and processed food products to “protect consumers from inferior products and to prevent ‘dumping' of imported substandard food” [(41), p. 21]. Research participants perceived that the Bahamian government was not doing enough to address the import of “export only” food products. “Export only” refers to food products that are not allowed by sold in the United States due to not meeting their regulatory standards. The Bureau's aims, therefore, appear to be in line with participant expectations, though their work is unknown to the participants.

In 2011, nutritional guidelines for public schools in The Bahamas were mandated through a set of School Healthy Food Standards. The standards have an aim of promoting “the principles and practices of good nutrition” [(46), p. 4], and build on prior policy responses, such as the Food-based Dietary Guideline of 2002. Foods high in fat, sugars, and oils are not to be sold. The standards are monitored by the Ministry of Education of The Bahamas but a health worker mentioned that there is no active monitoring and evaluation currently in place. Similarly, a community member who had kids in both private and public schools referred to the challenge of enforcement:

“*They [schools] are very strict when it comes to the snacks that we're allowed to send. But the food that they sell is contrary to this belief that you need to send healthy snacks.”*

The community member's quote suggests that there is a monitoring of foods brought into schools from home, but that the schools themselves are still selling unhealthy food items—contradictory to the standards. The quote also suggests that there is uptake in private schools despite the standards only being mandated for public schools.

In 2016, the Food Safety and Quality Act was passed to regulate food safety and increase capacity building to “ensure adequate policing of the food industry,” including through “collaboration and cooperation between public and private sector institutions” [(41), p. 21]. The Act similarly ensures labelling and advertising of foods are accurate and properly identifiable (e.g., through its name, dates of manufacture/expiration) and that businesses properly train staff to safely handle food.

In 2017, The National Multi-sectoral Non-Communicable Disease Strategy and Plan of Action for The Bahamas (2017 and 2022) was created and is considered “a response to the charge” given at the 2007 Port-of-Spain summit and is said to represent a commitment of the government, the Ministry of Health, civil society, the private sector and “corporate Bahamas” to “mount a national multi-sectoral strategy” to address NCDs [(47), p. 9]. This response, therefore, represents an instance where regional commitments directly resulted in policy action. It also is a recognition of the important role corporate Bahamas, or the business sector, plays in addressing factors related to obesity. The 2017 Plan recognises obesity as “by far the most pressing nutritional challenge facing The Bahamas” [(41), p. 8]. One of the key activities is lobbying for increased taxes on alcohol, tobacco, and sugar-sweetened beverages and subsidies on fresh fruits and vegetables. The lobbying activities contributed to the 2019 ban of sugar-sweetened beverages in public health and school facilities.

Similarly, in 2017, the National Food and Nutrition Security Policy and Agenda For Action (NFNSP) was created. It is a wide-ranging food policy with the vision of all people having “physical and economic access to sufficient, safe, and nutritious food” to meet their dietary needs and preferences for an active and healthy life [(41), p. 8]. The policy, therefore, addresses essential components of the food system, including food accessibility, availability, and affordability. These components have been linked to poor nutritional health outcomes in the Caribbean (48, 49). The NFNSP Policy builds on other nutrition-related policies, including the 2008 and 2010 Food and Nutrition Policy and Action Plans. The policy also mentions international and regional contexts as influential to the policy response. The country's commitment to CARICOM's Regional Food and Nutrition Security Policy and the Community of Latin American and Caribbean States (CELAC) plan to eradicate hunger by 2025 were important in recognising the role of poverty, social exclusion, and lack of participation in policy creation as causes to food and nutrition insecurity. The National Development Plan also provided an enabling environment as it set goals for achieving food security and improving nutrition for all.

In 2018, Farmers Markets launched in The Bahamas with “just under 100 active farmers in the program” (50). By the end of the year, the program was said to have about 220 farmers involved. The markets include farmers from across the archipelago, reaching farmers in the Out Islands as well. Out Islands are islands other than the capital island of New Providence (see Figure 2). It gives these Out Island farmers opportunities to sell “always Bahamian-grown… high-quality products at affordable prices” products directly to Bahamians at different pop-up locations, including in more urban islands like New Providence and Grand Bahama (50).

In December 2019, the Ministry of Health enforced that no sugar-sweetened beverages are to be sold or made available at public hospitals, clinics or health councils. The policy also applied to public schools (51), but compulsory standards which had banned sugar-sweetened beverages from schools have existed since 2011. This policy, therefore, supplemented this ban.

### Summary of key actions and activities since 2000

The identified policy responses broadly covered six areas (see Supplementary material 2). These areas include: (i) nutrition promotion; (ii) community engagement; (iii) combined approaches; (iv) advocacy; (v) food quality improvement; and (vi) other responses.

Nutrition promotion activities may be defined as interventions aimed at increasing healthy nutritional practises. It stemmed from the 2002 dietary guidelines which were used to promote healthy nutritional practises in the population. These efforts were supported by the 2011 Healthy Food Standards in schools where the guidelines were used to promote nutrition practise while limiting access to other foods through public and school engagements. Policy responses later moved to increase educational opportunities about nutrition through the launch of the Bahamas Agricultural and Marine Sciences Institute (BAMSI), in 2014, which also boosted the agricultural sector to increase access and availability of healthier food items in the country.

Community engagement interventions are those where policy responses took place directly in communities. Interventions included seminars in schools and workplaces, health fairs in shopping and community centres, and fun run walks or marathons. For example, community health nurses would demonstrate the amount of sugar in different food products in schools or workplaces to promote better nutritional practises. Another example is body mass index and blood pressure screenings at popular shopping centres and grocery stores.

Combined approaches are policy responses which integrated nutrition promotion with other interventions, including increasing physical activity. These combined approaches are more comprehensive as they focused on healthy lifestyles more broadly.

Advocacy was a noted policy response theme. These include lobbying activities for both specific policies (e.g., food labels) and groups of policies (e.g., healthy public policies). It was unclear the extent these lobbying activities went, but they may indicate a need to actively engage with stakeholders of importance.

Food quality activities focused mostly on food product safety and consumer knowledge. These interventions include ensuring that products are genuine and safe for consumption. For example, the Food Safety and Quality Act planned to ensure that food labels were standardised and that the advertisement of food products was accurate.

There is also a general “Other” category which included indirect interventions. For example, there was a noted need to boost the agricultural sector to address concerns about The Bahamas' high dependency on food imports (52). These imports are believed to be energy-dense and highly processed (15). Other responses included reducing smoking prevalence rates, improving health services and addressing Value Added Taxes (VAT) to make food, and the cost of living, cheaper (53). There was also an initiative to monitor healthy lifestyle programming nationally with the 2005 Healthy Lifestyle Initiative, but findings or reports of this activity were not identified.

### Key enabling and constraining factors for policy responses to obesity

Based on our analysis of interviews and review of documents, we found several constraining and enabling factors which influenced the public health policy responses to obesity in The Bahamas.

#### Notable constraints to policy responses to obesity

There were five notable constraints to the policy responses:

1. Political and multi-sectoral integration challenges
2. Gaps in legislative mandates
3. Need for monitoring implementation and updating policy
4. Funding and gaps in financial support
5. Sex and gender now coming into focus.

#### Political and multi-sectoral integration Challenges

The draft 2016 National Development Plan was developed with the Inter-American Development Bank (IDB), the University of The Bahamas, The Bahamas Chamber of Commerce, and government ministries. It has a steering committee which includes members from various government ministries including representatives in financial services, labour, tourism, youth, economy, governance, and Out Island development. Its consultation process included the public and private sectors. However, The NDP notes that a lack of buy-in from all sectors of society is a key challenge. These challenges may specifically be associated with issues related to private-sector profits. A health worker mentioned:

“*When you bring all the stakeholders to the table I think that's where the issue come in because we gotta deal with the wholesalers, this and that, they not willing to take that hit on all fruits and vegetables so they tryna be selective with certain ones. But, what can you say?...They have a tremendous influence because money, money talks. When you have money you have power, you have more influence. It talks. So like I said, if it was up to us [health workers] all fruits and vegetables would be on that breadbasket list. The mere fact that all fruits and vegetables at the moment are not on it speaks to the level of corporate influence.”*

The health worker believes that the food industry hampers reform processes to make fruits and vegetables more affordable as it can undermine their profits. A policymaker interviewed similarly referenced the balance of power between the Ministry of Health and other actors, mentioning that the Minister of Health “*is just one of many others in Cabinet*.”

The participants are recognising dynamics that different groups have different kinds and levels of power (54). Therefore, despite it being a common strategy to include all stakeholder groups in health policy reforms (55, 56), it does not necessarily mean that all interests are aligned, which can prove challenging when policy responses depend on multi-sectoral collaboration.

Political challenges also arose. There were concerns that a new government administration would not prioritise the implementation of the plan. This led the then Investments Minister to urge the next government administration “not to interfere with the National Development plan's crafting,” and advised on the non-partisan nature of the NDP (57). All three major political parties of The Bahamas are listed as “Stakeholders and Partners” of the NDP (58).

In 2019, Chamber of Commerce executives, one of the stakeholders of the plan, expressed concerns that the NDP had “gone cold”—meaning that there was no action on its implementation (59). Hartnell (59) notes that the new government administration made “virtually no mention” of the NDP once taking office, raising concerns about the fate of the plan. Sutton (60) observes that in Caribbean states, high-level specific policies are often associated with particular officials, or parties, which may lead to policy failure once that official, or party, is out of office. This means that policies which address the prevalence of obesity may be shelved if associated with members of the opposing party.

This has implications for the timeline of policy implementation and resource use due to replicated efforts.

#### Gaps in legislative mandates

Some policy responses were constrained by the lack of legislative backing, which means that some policy responses were side-lined. A need for the enforcement of the 2002 Dietary Guidelines was noted by a policymaker who mentioned that the Guidelines “*need to be revised*,” calling them “*principles*” while noting the gaps in the mandate. This was similarly echoed by BMOH and BMOAMR (41) who called for further expansion of the Guidelines, universal enforcement, and greater consistency of the Guidelines with other policies. This would ensure that the policy is consistent across other policy responses and provide additional support for the Guidelines' aim to support healthy eating habits among Bahamians.

Similarly, The Bureau of Standards and Quality hopes to adopt front-of-package and nutrient labelling standards for all food products in the country. However, it is still unclear how far these labels will go. In Mexico, consumers were faced with ominous black labels on food and beverages which warned if they contained too much sugar, fat, or calories (61). The move prompted legal challenges from the food industry in Mexico.

Participants opined that, currently, nutrition labels are not used by Bahamians. A health worker said she does not see Bahamians currently using labels to make decisions about what products to buy. This was corroborated by a community member who mentioned that Bahamians “*don't pay attention*” to it. The community member indicated that food labels are not used because the names of different ingredients (she used the word “*chemicals*”) are not accessible to people and that the “*chemical names are intimidating*.” The community member perceived that changing the labels to be colour coded would increase costs for Bahamian companies who “*would have to compete with foreigners*” and therefore the incentive to do so is not there. She expressed that it was “*difficult from an economic standpoint*.” The health worker also noted that “*we don't produce our own food no way*” so such a measure may not be relevant. The comments refer to the fact that The Bahamas imports the majority of its food.

The health worker also spoke about the sociocultural challenges of implementing new nutrition labels. She opined that she does not think the black labels, to represent the high content of foods, would work in The Bahamas because some Bahamians associate black with voodoo. This speaks to the majority of Bahamians following the Christian religion and a belief that voodoo is unchristian. Both the community member and health worker believed that more education was needed about food labels. However, another community member opined that the government “*should push it forward*” as it is a useful tool for “*becoming more conscious*” about healthy eating.

These examples detail that policy responses can be limited by these gaps in legislation enforcement as some policy responses are side-lined.

#### Need for monitoring implementation and updating policy

A lack of active monitoring and evaluation of policy responses leave some policy responses in need of revision. Despite being credited as the first to have localised food-based dietary guidelines in the Caribbean region, the 2002 guidelines are in need of revision. A health worker noted that the guidelines are “*supposed to be done every 10 years*,” but an update was not identified. Another health worker indicated that the government is not “*consistent with following up or implementing or yes, monitoring and evaluating the situation…we're not as active as we should*.” The guidelines appear not to be used widely due to a lack of awareness of their existence. Only one policymaker referenced the guidelines and a community member told me that she would recommend the country have its own “*healthy food plate*”—a reference to the food-based guidelines, which already exist but that she was not aware of. According to BMOH and BMOAMR (41), there are plans to undertake a media campaign between 2017 and 2022 to promote the guidelines with a budget of $50,000 led by a National Food and Nutrition Coordinating Commission. The Commission would be jointly chaired by the Ministers of Health and Agriculture and include members of the public, private sector institutions, and NGOs.

One reason for this gap in monitoring and evaluation mechanisms may be that countries in the Caribbean face strained human and financial resources (62, 63), which limit health system capacity. These settings must also contend with multiple demands and priorities from international donor organisations which absorb human and financial resources (64).

Nonetheless, the need for active monitoring policy responses is a noted challenge.

#### Funding and gaps in financial support

Most policy documents reviewed did not explicitly mention funding sources. Where financial support was mentioned, it can be categorised into three main sources: (i) the MOH, (ii) other Bahamian government ministries (i.e., the Ministry of Finance, Ministry of Agriculture and Marine Resources and Ministry of Social Services), and (iii) international organisations (i.e., PAHO and the Inter-American Development Bank).

Several policy responses were limited by gaps in financial support, particularly those focused on public outreach interventions. It is often the case that MOH budgets do not adequately finance preventative and health promotion activities at the same level as curative services (21). For example, the 2005 Healthy Lifestyles Initiative, which organised events within the community (e.g., health screenings, health/nutrition advice), was said to be limited by a lack of financial support and a call was made for increased funding for preventative health care and “healthy lifestyle promotion” programmes, such as the Initiative [(21), p. 30].

Similarly, The Healthy Bahamas Coalition notes that one of its key challenges is “inconsistent funding streams due to competing national priorities” (65). The Coalition is financed through the MOH budget and PAHO country funds but also seeks donations. It notes that it “receives no funds from fast-food enterprises, sellers of high fat, high salt, high sugar foods, alcohol companies, tobacco sellers, and gambling houses” (65). This was the only programme which explicitly stated a conflict of interest policy. A policymaker mentioned that the Coalition has “*needed support for 2 years*”—a further indication that the Coalition does not have sufficient funds to fulfil its mandate successfully. We found, however, that the Coalition is still actively leading community engagement activities, including hosting expert speaker events virtually.

A policymaker mentioned that the MOH is prepared for further comprehensive policy responses, but that some interventions require coordination and funding from/with other sectors.

#### Sex and gender now coming into focus

More recent policy responses (i.e., from 2016) increasingly focused on gender and recognised its importance in addressing health issues. The draft 2016 National Development Plan, for example, recognises women's societal and labour roles in The Bahamas as an important determinant of health. The 2017 NFNSP policy recognises women's historical importance to food production needs in the country, including in household subsistence farming (8), and the need to provide additional resources in line with their importance. It also recognises women as a marginalised group, particularly in entrepreneurship. There is scope, however, to also address other sociocultural components of gender, including factors related to cultural beauty ideals which promote larger body sizes (particularly for Black Women) (23), advertisements which specifically target women and children (4), and gendered attitudes that promote unhealthy lifestyles (66). These interventions will require the support of the non-health sector. Interventions to address the gender health disparity in the prevalence of obesity should also be integrated into all policies and not handled as an add-on via a separate policy (67).

In sum, these five notable factors may be linked to increasing or sustaining obesity prevalence rates by constraining policy responses. Political and multi-sectoral integration challenges can elongate the timeline for policy responses and/or duplicate efforts. Gaps in legislative mandates mean that enforcing practises to address obesity prevalence can be side-lined. A need for active monitoring of policy responses means that policies are not updated to address emerging contextual factors. Meanwhile, gaps in financial support limit community engagement activities. Sex and gender are now coming into focus, a step in the right direction. However, there was a gap in earlier policy responses. These constraints have implications for the prevalence of obesity in the country.

#### Notable enabling factors for policy responses to obesity

There were also enabling factors that facilitated policy responses. These factors included:

1. Building on consistent policy responses
2. Multi-sectoral support and collaboration
3. Buy-in for further action.

#### Building on consistent policy responses

Between 2000 and 2019, there have been consistent policy responses to address nutrition and health outcomes, including obesity. These policy responses increasingly built on each other to address previous gaps and/or make newer policy responses more comprehensive.

For example, the 2005 Healthy Lifestyles Initiative led to an expansive 2016 Healthy Bahamas Coalition. The Coalition has 12 subcommittees working toward addressing different aspects of its objectives (including nutritional outcomes, policy advocacy, and healthy lifestyles) and has membership from the public, private, and civil society sectors—including members from clinics, schools, government departments, health worker associations and unions, universities, banks, and nutrition consultants. This more expanded Coalition led to more integrated approaches, such as partnering with workplaces in the private sector. A policymaker noted that several other activities were also conducted, but were not recorded on paper for future reference.

There is also consistency in data collection. The Bahamas carried out the WHO STEPS surveillance multiple times, including in 2012 and 2019. Moss (68) reports that The Bahamas also carried out the survey in 2005, but the MOH did not label the 2005 survey as a STEPS surveillance. In 2005, The Bahamas carried out a CNCDs Prevalence Study and Risk Factor Survey in which the country conducted the first two “STEPS” of the WHO's Approach. These STEPS were a questionnaire and physical measurements of individuals. The third STEP calls for biochemical measures (69). A policymaker saw it as a source of pride that “*The Bahamas is one of the only countries*” to have completed the STEPS Surveys “*in its entirety*.” Moss (68) reports that The Bahamas conducting multiple rounds of the STEPs surveys is “a reflection of the political and attending financial commitment to advance evidence-based policies of wellness and health system improvement.” The Minister of Health indicates that the surveys help to “tell us about the health of our nation, forecast and plan for the future demands for health services related to NCDs” (68). Therefore, the surveys provide evidence for future policy actions and allow for further consistency in policy responses.

#### Multi-sectoral support and cooperation

While there were political and multi-sectoral challenges, there were instances of cooperation. Policy responses became increasingly comprehensive as they worked across multiple sectors beyond the health sector (e.g., agriculture, private sector). This leads to addressing determinants of health that the health sector alone is unable to do.

The 2017 National Multi-sectoral Non-Communicable Disease Strategy and Plan of Action for The Bahamas is perhaps among the most comprehensive as it prioritises mounting a multi-sectoral strategy to address nutrition and health outcomes. It is a commitment of the government, MOH, civil society, private sector, and corporate Bahamas to work together in addressing factors related to obesity. These factors include strategies to address nutrition including taxing unhealthy food items and sugar-sweetened beverages while reducing the costs of healthier food items, including fruits and vegetables.

Similarly, the 2017 National Food and Nutrition Security Policy and Agenda For Action (NFNSP) policy established a multi-sectoral committee to “review the current food, nutrition and health situation and set guidelines for improving food and nutrition security for all segments of the population” and tourists (44). The development of the 2017 NFNSP policy has been lengthy and required frequent advocacy and commitment. A comprehensive nutrition security plan was first advocated for in 1991 by the MOH. A policymaker mentioned that they took a strategic approach to bring other sectors on board for more comprehensive and multi-sectoral policies by arranging individual meetings with each stakeholder to hear and respond to concerns, including private sector grocers.

These more multi-sectoral policies are therefore more comprehensive and have broad support from multiple sectors, at least initially.

#### Buy-in for further action

Participants interviewed generally supported policy responses, including making some responses more robust by further action and closing loopholes.

The government's approach to banning sugar-sweetened beverages in public health and education facilities was among the most widely discussed policy response by research participants. This may be, in part, because the response was implemented during the time of fieldwork (i.e., December 2019). The policy response appears to have had broad, though not universal, support. An online reader poll of 143 readers of The Tribune newspaper in The Bahamas showed that only 12% did not support the move (51). A research participant believed the step to ban sugar-sweetened beverages from public facilities was “*very small*” and he perceived that “*it's more a principle statement*.” Nonetheless, he praised the MOH for acknowledging that “*this shit is killing you*.” A health worker similarly mentioned that the move was a principled start and called for a stricter response because “*people still have a lot of access to it*.” Therefore, there is support for such policy responses to extend further by limiting access to sugar-sweetened beverages even beyond these public facilities.

There was also support for making healthier food options more affordable as there was a perception among research participants that healthy foods were more costly. A health worker mentioned that the “*VAT thing going on*” (i.e., value-added tax) has dissuaded healthy eating, particularly in a context where salaries have not increased. The health worker asked rhetorically, “*How is it that you want me to eat healthy?...I gotta eat what I could eat*.” This is a reference to Bahamians eating foods that they could afford and the need to do more.

In sum, these three factors may be linked to reducing or limiting obesity prevalence rates by facilitating policy responses. Policy responses increasingly built on each other to address previous gaps and/or make newer policy responses more comprehensive. Multi-sectoral support and collaboration also facilitated policy responses that were more comprehensive by including areas outside of the health sector. This leads to addressing more determinants of health, which the health sector alone is unable to do. There was also buy-in for further action to make current policies more robust. This support may facilitate future responses.

## Discussions

A series of health policy responses since 1970 demonstrated an evolution of nutritional health challenges in The Bahamas. The Bahamas has largely been successful in addressing undernutrition in the country, but grappling with rising rates of obesity linked to overconsumption of unhealthy foods is now of concern.

**Figure 5:**
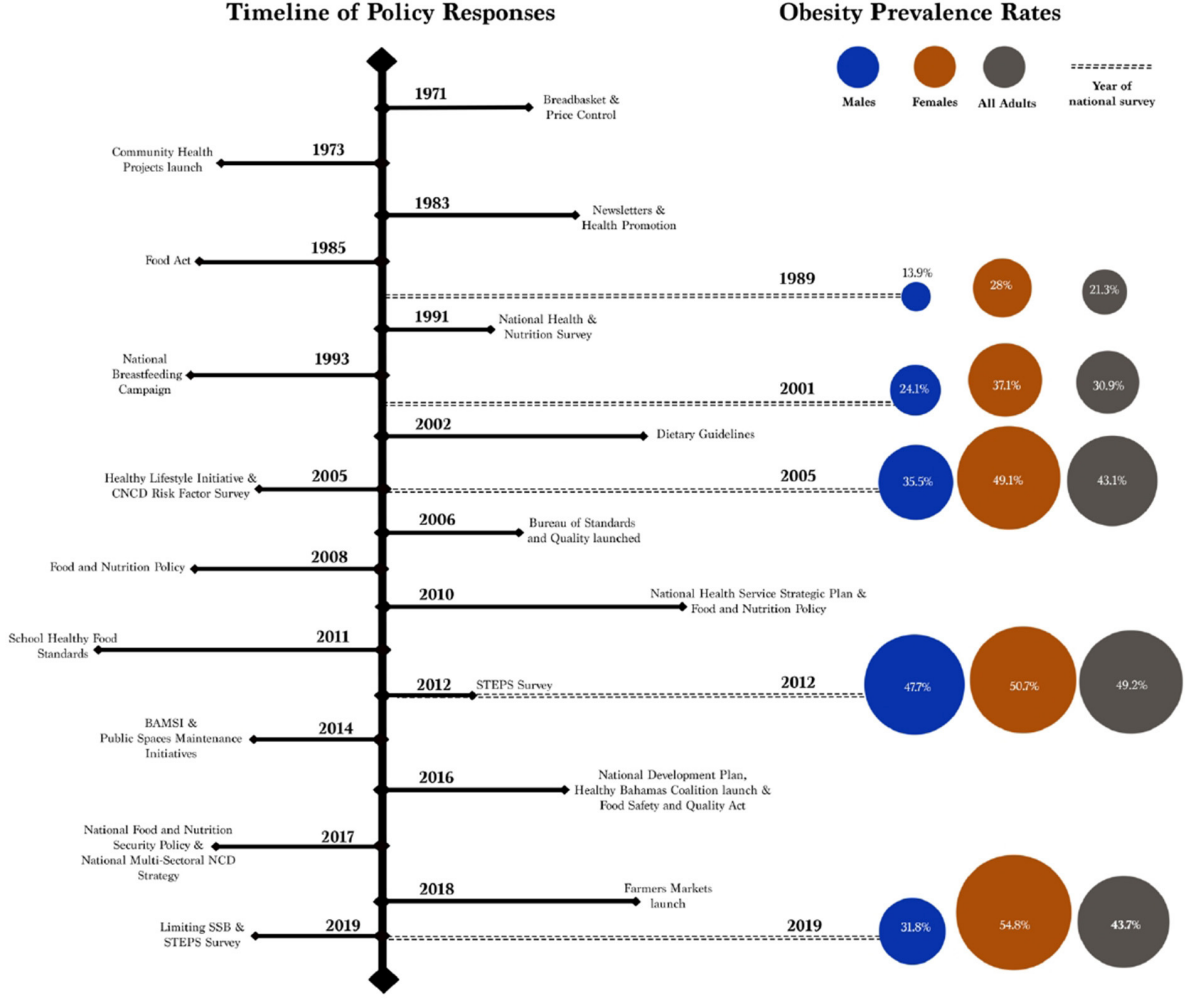
Time of policy responses and obesity prevalence rates in The Bahamas between 1971 and 2019. The figure shows that policy responses may have led to a decrease in the prevalence of obesity between 2012 and 2019. There is, however, a gendered health disparity where the prevalence continued to increase in women during the period. This indicates to make additional progress, there is room for further interventions that consider the gender components that influence the prevalence of obesity.

According to national reports reviewed, obesity prevalence rates increased up to 2012 when the prevalence rates reached 49.2% in the adult population before a decline to 43.7% in 2019. This represents a reversal of trends in the prevalence of obesity given that there has been a continued increase since 1989. This suggests that national policies, population-level interventions, and community engagement activities may have been effective in reversing over 20 years of increases in obesity prevalence (see Figure 5). These actions and activities included nutrition promotion; community engagement; combined approaches between nutrition, physical activity, and health promotion; advocacy for healthy public policies; addressing food standards and quality; and other interventions like improving the agricultural sector, addressing taxes, and more. Addressing the prevalence of obesity at multiple levels is more comprehensive and results in more sustainable approaches (70, 71).

Factors such as sustained implementation of policy responses using multi-sectoral support and collaboration, and obtaining buy-in from the community are key enabling factors for reducing or limiting obesity prevalence. These were especially exemplified in more recent policy responses (i.e., since 2016). For example, the 2017 National Food and Nutrition Security Policy, one of the most comprehensive policies identified, used a combination of approaches to increase educational opportunities about nutrition, engage the public about nutrition, lobby for healthy public policies, increase access and availability to healthy foods, and boost the agricultural sector. These activities build on previous policy responses (e.g., the launch of The Bahamas Agricultural and Marine Sciences Institute in 2014), included multiple sectors (e.g., Ministry of Agriculture) and obtained buy-in from the community (e.g., community engagement interventions). These three factors would be consistent with best practises recommended by the Caribbean Public Health Agency (CARPHA) to address obesity and other NCDs (72). It also emphasises the importance of a sustainable long-term strategy in making progress on obesity prevalence and other NCDs (3).

There is, however, a divergence of trends along gender lines. While the prevalence rate among men dropped between 2012 and 2019, it increased in women during the same period. These data are consistent with global literature where it is observed that the prevalence of obesity is higher in women (4, 73). Gender relations may therefore be a factor. Gender relations in the social, economic, and cultural environment can combine and affect health status (66), including obesity (4). More examination of gender relations in the Bahamian context is therefore needed to determine its impact on obesity prevalence, despite policy responses. Further contextual analysis can also add to our findings, given that in the Caribbean, cultural attitudes on body weight and shape, the influence of the media, peer influence, exercise, and eating habits can impact obesity prevalence (4). These gendered and sociocultural influences may be implicated in the sustained increase in obesity prevalence rates up to 2012, including along gender lines.

There are also other challenges and risks to progress identified from 2012 to 2019. Public health policy was stalled by political ideology in at least one case, such as the draft 2016 National Development Plan. Political ideology can undermine high-profile policy interventions as new government administrations move to overturn previous administration advances (60). Politics can therefore elongate policy implementation timelines and threaten consistency in policy responses to obesity. There are also challenges to multi-sectoral collaboration as priorities can be different across sectors and/or actors. This can elongate legislative processes. CARPHA acknowledges the need for the whole of government and whole of society approaches to address health and nutritional outcomes (4). This emphasises the need for multisector collaboration, despite these challenges.

There were also noted gaps in monitoring the implementation progress of different policies and in financial support for programmes—particularly those related to community engagement. According to the Healthy Caribbean Coalition (HCC), community-level interventions are critical in preventing and controlling obesity (15). There is a need to ensure programmes, particularly at the community level, are adequately funded and sustained to achieve policy goals.

## Limitations

The study has two key limitations. First, public health policy responses were not contextualised into the wider socio-cultural system in which the health system functions. This would have allowed for the incorporation of micro- and meso-level detail about the cultural constructions of obesity and issues of gender intersections. This limitation can also be used to inform further policy action. Second, our research looked specifically at policies from the health sector. This limitation meant that policies that non-health sectors may have led in The Bahamas were excluded from this study. Future research can build on the findings of this research by widening the scope of policy analysis to include policies from other sectors (such as the Department of Information, Ministry of Agriculture, Ministry of Education etc.) that may have implications for population health.

## Conclusion

The findings of this paper have shown that recent progress manifesting from sustained multilevel policy responses and population-level interventions are effective in reversing over two decades of continued increase in obesity prevalence rates in The Bahamas. However, there is a need to redouble efforts and implement further gender-specific and sociocultural responses to maintain the downward trend in the prevalence of obesity while improving universal access to nutritious food by increasing its availability and affordability for all. This would accelerate the achievement of several Sustainable Development Goals in The Bahamas, including Goal 1 on ending poverty, Goal 2 on ending hunger and improving nutrition, Goal 3 on ensuring healthy lives and improving well-being, and Goal 5 on gender equality. Finally, whilst the prevalence of obesity has decreased in the country between 2012 and 2019, future research should explore the socio-cultural and political-economic factors that contributed to this reduction alongside policy responses.

## Data availability statement

The original contributions presented in the study are included in the article/Supplementary materials, further inquiries can be directed to the corresponding author.

## Ethics statement

The studies involving human participants were reviewed and approved by University of Leeds School of Medicine Research Ethics Committee and Public Hospital Authority/University of the West Indies Research Ethics Committee in The Bahamas. The patients/participants provided their written informed consent to participate in this study.

## Author contributions

FP conceived the study. FP and BE conceptualised the article. FP led the writing of this paper with contributions from BE and RK. All authors contributed to the article and its revisions, and approved the submitted version.

## Conflict of interest

The authors declare that the research was conducted in the absence of any commercial or financial relationships that could be construed as a potential conflict of interest.

## Publisher's note

All claims expressed in this article are solely those of the authors and do not necessarily represent those of their affiliated organizations, or those of the publisher, the editors and the reviewers. Any product that may be evaluated in this article, or claim that may be made by its manufacturer, is not guaranteed or endorsed by the publisher.
