# Supplementary material for: Impact of sustained health policy and population-level interventions on reducing the prevalence of obesity in the Caribbean region: A qualitative study from The Bahamas
Source: Front Public Health. 2022 Aug 30;10:926672. doi: 10.3389/fpubh.2022.926672 (PMC9468752; doi:10.3389/fpubh.2022.926672)
Supplement: Supplementary file 1 [file Table_1.docx]

**Supplementary Material 1**

## Key documents reviewed

| **Year** | **Name** | **Developed by** | |
| --- | --- | --- | --- |
| Various | MOH News Newsletter (1989 – 1990) | MOH Bahamas |  |
| Various | Breadbasket Food Items and Price Control | Bahamas Parliament |  |
| Various | Annual Reports of the Chief Medical Officer (1975 – 1984; 2000; 2001 – 2003; 2004 – 2008) | MOH Bahamas |  |
| 1983 | Joining Hands for Health Newsletter | Health Education Division of MOH Bahamas |  |
| 1985 | Food Act | Bahamas Parliament |  |
| 1991 | National Health and Nutrition Survey (1988 – 1989) | MOH Bahamas, Caribbean Food and Nutrition Institute, PAHO/WHO |  |
| 1998 | Bahamas Youth Health Survey | MOH Bahamas |  |
| 1999 | A New Vision for Caribbean Health | Caribbean Cooperation in Health Secretariat |  |
| 2000 | WHO Global Strategy for the Prevention and Control of Chronic Diseases | WHO/PAHO |  |
| 2002 | Food-based Dietary Guidelines | MOH Bahamas |  |
| Various | Health in The Americas Reports and Bahamas Profile (2002; 2017) | PAHO |  |
| 2004 | Bahamas Living Conditions Survey 2001 | Department of Statistics |  |
| 2004 | Global Strategy on Diet, Physical Activity and Health | WHO |  |
| 2006 | Chronic Noncommunicable Diseases: Identifying Determinants to The Bahamas’ Burden | MOH Bahamas |  |
| 2006 | Regional Strategy and Plan of Action on an Integrated Approach to the Prevention and Control of Chronic Diseases including Diet, Physical Activity and Health (CD47/17, Rev. 1) | PAHO |  |
| 2007 | Port-of-Spain Declaration | CARPHA |  |
| 2010 | National Health System Strategic Plan 2010 – 2020 | MOH Bahamas, Public Hospitals Authority |  |
| 2011 | Population-based and Individual Approaches to the Prevention and Management of Diabetes and Obesity | 2011 |  |
| Various | The Bahamas STEPS Survey (2012; 2019) | MOH Bahamas |  |
| Various | State of Public Health in Caribbean region (2013; 2017; 2019) | CARPHA |  |
| 2014 | Intervention by Hon. Philip E. Davis M.P Deputy Prime Minister and Minister of Works & Urban Development Commonwealth of The Bahamas | Philip Davis (MP) |  |
| 2016 | Household Expenditure Survey 2013 Report | Department of Statistics |  |
| 2016 | State of the Nation Report | National Development Plan Secretariat |  |
| 2016 | National Development Plan (Draft) | National Development Plan Secretariat |  |
| 2017 | Preventing Childhood Obesity in the Caribbean Civil Society Action Plan 2017 – 2021 | Healthy Caribbean Coalition |  |
| 2017 | National Food and Nutrition Policy and Agenda for Action | MOH Bahamas, Ministry of Agriculture and Marine Resources |  |
| 2017 | National Multi-Sectoral Non-Communicable Disease Strategy and Plan of Action for The Bahamas (2017 – 2022) (Executive Summary) |  |  |
| 2017 | Promoting Healthy Diets, Food Security and Sustainable Development in the Caribbean through joint Policy Action | CARPHA |  |
| 2018 | National Statement High-Level Political Forum on Sustainable Development United Nations Economic and Social Council (ESOSOC) | Hubert Minnis (MP) |  |
| 2019 | NCDs at a Glance: NCD Mortality and Risk Factor Prevalence in the Americas | PAHO |  |
