# Supplementary material for: Impact of sustained health policy and population-level interventions on reducing the prevalence of obesity in the Caribbean region: A qualitative study from The Bahamas
Source: Front Public Health. 2022 Aug 30;10:926672. doi: 10.3389/fpubh.2022.926672 (PMC9468752; doi:10.3389/fpubh.2022.926672)
Supplement: Supplementary file 2 [file Table_2.docx]

**Supplementary Material 2**

**Summary of key actions and activities from policy responses in The Bahamas since 2000**

| **Policy** | **Key actions and activities** | | | | | |
| --- | --- | --- | --- | --- | --- | --- |
|  | **Nutrition Promotion** | **Community Engagement** | **Combined Approach** | **Advocacy** | **Food Quality** | **Other** |
| Food-based Dietary Guidelines (2002) | Guidelines to promote healthy nutrition practice |  |  |  |  | New funding to promote awareness of guidelines |
| Healthy Lifestyle Initiative (2005) |  | Workplace, school, community engagements (e.g. risk factor screenings) | Targets nutrition and physical activity increase |  |  | Reducing prevalence of smoking  Supporting research on behaviour modification |
| Bureau of Standards and Quality (2006) |  |  |  |  | Standardisation of food labels and products |  |
| National Health Service Strategic Plan (2008 & 2010) |  | Engaged NGOs, public, private sector to address their concerns |  |  |  | Aimed to improve health services generally |
| Food and Nutrition Policy (2008) | Promote nutrition awareness |  |  |  |  |  |
| School Healthy Food Standards (2011) | Promote nutrition awareness | Public and school engagements |  |  |  |  |
| Bahamas Agricultural and Marine Sciences Institute (2014) | Increase educational opportunities about nutrition | Engagements with public, schools and private sector actors |  |  | Increase access and availability of healthy foods |  |
| Public Spaces Maintenance Initiatives (2014) |  |  | Nutrition and physical activity |  |  | Restores access and maintains public spaces for organized events |
| National Development Plan (2016) |  | Public engagements via consultations | Nutrition, physical activity and health promotion approaches | Lobbying for healthy public policies | Increase access and availability of healthy foods | Boosting agricultural sector |
| Healthy Bahamas Coalition (2016) | Promote nutrition awareness | Workplace, school, community engagements | Nutrition, physical activity and health promotion approaches | Strategic planning to address or advise nutrition policy |  |  |
| Food Safety & Quality Act (2016) |  | Training for staff in workplaces that handle food |  |  | (a) Standardisation of food labels and products  (b) Policies on accuracy of food advertising |  |
| National Food and Nutrition Security Policy (2017) | Increase educational opportunities about nutrition | Engagements with public, schools and private sector actors | Combined nutrition and health promotion | Lobbying for healthy public policies | Increase access and availability of healthy foods | Boosting agricultural sector |
| National Multi-Sectoral NCD Strategy (2017) | Promote nutrition awareness | Engagements with public and private sector actors | Combined nutrition, physical activity and health promotion approaches | Lobbying to:  (a) increase taxes on alcohol, tobacco and SSB  (b) subsidies for fresh fruits and vegetables |  |  |
| Farmers Market (2018) |  | Various pop up markets close to communities |  |  | Increase access and availability of healthy foods |  |
| Limiting consumption of Sugar Sweetened Beverages (2019) |  |  |  | Blocking SSB consumption in public health and education facilities |  |  |
